# Development and Psychometric Properties of the Community Implementation Behaviour Questionnaire (CIBQ) in the Context of Supporting Caring Relatives of People with Dementia

**DOI:** 10.3390/ijerph192316198

**Published:** 2022-12-03

**Authors:** Maren Wittek, Fabian Manke-Reimers, Eric Schmitt

**Affiliations:** 1 Institute of Gerontology, Faculty of Behavioural and Cultural Studies, Heidelberg University, Bergheimer Straße 20, 69115 Heidelberg, Germany; 2 Center for Preventive Medicine and Digital Health, Medical Faculty Mannheim, Heidelberg University, Ludolf-Krehl-Straße 7-11, 68167 Mannheim, Germany

**Keywords:** implementation science, municipal community, caring relatives, people with dementia, support services, theoretical domains framework, psychometric properties

## Abstract

The Theoretical Domains Framework (TDF) investigates the determinants influencing the implementation behaviour of actors in healthcare. Caring for people with dementia (PWD) can be burdensome. Therefore, caring relatives (CRs) often rely on support of various actors in their community (CAs). However, the support of this target group is not sufficient, and the implementation of support services needs to be optimised. As it stands, there is no German-language questionnaire to investigate the factors that influence the implementation behaviour of CAs. Therefore, based on the TDF, the Community Implementation Behaviour Questionnaire (CIBQ) was developed in this study. A total of 205 CAs from 16 German communities were surveyed. The 34-item CIBQ asked about their implementation behaviour regarding support services for CRs of PWD. To identify the best model fit, the internal consistency and construct validity were computed. After adaptation, the final CIBQ consisted of ten domains and thirty-one items. The psychometric properties of the questionnaire are as follows: CMIN/DF = 1.63; SRMR = 0.05; RMSEA = 0.07; CFI = 0.92; Cronbach’s alpha 0.74–0.89; inter-item correlation 0.38–0.88. The initial results show satisfactory internal consistency and construct validity of the CIBQ. Using the CIBQ enables the health and care optimisation of CRs of PWD.

## 1. Introductions

Adequate procedures in the implementation process are required for the successful long-term implementation of evidence-based interventions in standard care [1,2]. The Consolidated Framework for Implementation Research by Damschroder et al. (2009) and the Theoretical Domains Framework (TDF) by Michie et al. (2005) are two examples of strategies and theories for designing and controlling the implementation process and can be used to understand the behaviour of healthcare professionals within such processes [2,3,4,5].

The present study was concerned with the TDF as it addresses the implementation behaviour of different actors and their characteristics [5]. Much is known about the structural determinants of implementation processes, such as time and money constraints [6,7]. Furthermore, according to scientific findings, the person-related factors of the actors also play an important role in the implementation of measures or in explaining suboptimal implementation. These include, for example, their knowledge, their professional role or their motivation [8]. However, the impact of these personal characteristics needs to be examined more closely in different contexts. Personal characteristics are covered in the TDF, which makes this framework particularly well-suited for research in the field of behaviour change in implementation processes [5,9]. Michie et al. (2005), along with several health researchers, generated this validated theoretical framework for implementation science by using an extensive consensus process [5]. The TDF answers the question of which characteristics of actors influence their behaviour during the implementation of interventions and consists of twelve domains, including knowledge, beliefs about capabilities and social influences [5]. The TDF has been used as a basis for many quantitative questionnaires (e.g., [10,11]) and qualitative research (e.g., [12,13]) on different topics within the healthcare sector. Therefore, the TDF has often been adapted to the context of the respective topic. However, to our knowledge, there is no suitable tool for the gerontological context, especially in connection with the responsibility of actors from municipal communities.

One gerontological topic for which the application of the TDF would be relevant is the support of caring relatives (CRs) of people with dementia (PWD) within their municipal community. It is understood from discussions and the literature that the extent to which actors from the community (CAs) support CRs of PWD is highly dependent on their individuality [14,15]. In Germany, communities, among others, are responsible for providing adequate support for CRs of PWD [16,17].

Worldwide, there are over 55 million PWD [18]. In Germany, there are currently about 1.7 million people living with dementia, of whom two-thirds are cared for at home by their relatives [17,19]. Therefore, CRs are an important pillar of the German care system and are deserving of support [16]. However, the degree to which communities implement support services for CRs who are physically and/or psychologically burdened varies greatly [20,21,22,23]. Although caregiving for PWD often negatively impacts the health and daily lives of CRs [24,25] and numerous studies show that support services can improve the well-being and quality of life of this population, successful implementation often fails to occur [26].

The structural barriers to receiving support are partially understood [27,28], but there is a need for a framework and tool to examine potential implementation barriers with a focus on personality traits and behavioural change in the context of supporting CRs of PWD within municipal communities. Wittek et al. (2022) undertook a qualitative investigation in this context and highlighted the need for a quantitative tool to look more closely at such characteristics [29]. A quantitative questionnaire based on the TDF needs to be developed to enable a resource-efficient, timesaving and generalisable examination of the implementation behaviour of CAs, especially concerning the demands of CRs of PWD.

The purpose of adapting and developing the questionnaire is to answer the following question: which characteristics of CAs influence their behaviour during the implementation of support services for CRs of PWD in communities? To answer this research question, we developed and established an adapted tool: the Community Implementation Behaviour Questionnaire (CIBQ).

**Figure 1:**
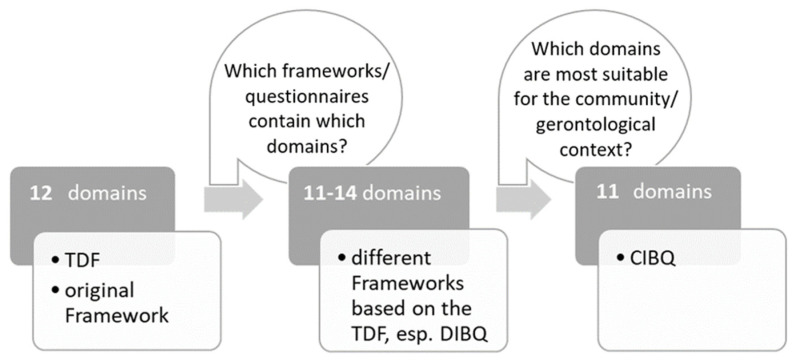
Adaption and selection process of the discussed domains. TDF = Theoretical Domains Framework; DIBQ = Determinants of Implementation Behavior Questionnaire; CIBQ = Community Implementation Questionnaire.

The adaptation and selection process of the domains discussed in this paper is described in the methods section and is presented in Figure 1.

## 2. Materials and Methods

The aim of the study was to develop a tool that can examine the characteristics of CAs that influence their behaviour during the implementation of support services for CRs of PWD in communities and to verify its psychometric properties. A cross-sectional observational study of actors from different communities in Germany was conducted.

### 2.1. Context

The recruitment of the study participants was carried out via a cooperation project called “Giving a voice to caring relatives of persons with dementia—The Town Hall Project” in which town hall meetings were held in communities in Germany. The Townhall meetings intend to achieve a dialogue between CRs of PWD and CAs from different work sectors in the community. The participants discussed the needs of CRs and possibilities for their support. Based on defined criteria such as region (urban and rural), number of inhabitants and accessibility, municipal communities were selected to receive an invitation to participate in the Town Hall Project. A total of 45 municipal communities were invited to participate, and 16 municipal communities finally participated. Communities that did not participate gave reasons such as difficulties in finding dates or the workload involved. In each community, a multiplier such as a nurse or a senior citizen counsellor established contact with CAs, which enabled us to recruit them for the town hall talk and/or participation in the cross-sectional observational study described in this paper. These actors were either already working with CRs and/or PWD or could potentially work with them. They were from different fields of work in the community, such as municipal administration, nursing, healthcare, voluntary work, consulting, church, sports, culture, education and housing.

The project was approved by the Ethics Committee of the University of Heidelberg, Faculty of Behavioural and Cultural Studies, in 2019.

The distribution of the 16 municipal communities within Germany and the methodological procedures of recruitment and other content-related aspects about the town hall meetings have been previously published [30,31]. In the present study, the town hall project was used exclusively for recruitment. The data collection and analysis were completely separated.

### 2.2. Development of the CIBQ

The CIBQ was initially based on the original version of the TDF, which consists of twelve domains of behaviour change [5]. With the inclusion of further existing quantitative questionnaires that are also based on the TDF [5,9,10,32,33,34,35], the framework was adapted for this study and the described context. The authors of these questionnaires had previously adapted, extended, and specified some of the domains and included between eleven and 14 domains in their work. For the CIBQ, many domains were compared, and the eleven most suitable domains were selected. The Determinants of Implementation Behavior Questionnaire (DIBQ) by Huijg et al. (2014) was considered to be the best fit [33].

Although the current study focused on the behaviour of implementing support services for CRs of PWD in the community, the adaptation of the domains and items could be readjusted and used for CAs within the gerontological context in general. For the development and adaptation of the CIBQ, a combination of evidence from the literature [5,9,10,32,33,34,35] and experts’ opinions (e.g., politicians at the federal and state levels, mayors, senior citizen advisors, volunteers, and scientists) were taken into account. First, the research team translated and modified the existing domains and items, with a particular focus on Huijg et al. (2014), before the experts gave their feedback. This approach ensured maximum construct validity. The results of this collaborative process, including the choice of domains and their definitions, are shown in Table 1.

As the frameworks and questionnaires utilised were written in English and the target group was German-speaking, the definitions and items needed to be translated. There is a procedure for producing accurate translations and adaptations of measures. The procedure includes the following five steps: (1) forward translation; (2) comparison of two translated versions and consensus meeting; (3) blind back-translation; (4) comparison of two back-translated versions and consensus meeting; and (5) pilot testing of the pre-final version and adaptation of the questionnaire [36,37,38]. As a result of the extensive adaptation of the domains and items, phases three and four were omitted [36,37,38].

The corresponding quantitative questionnaire was based on self-completion and self-report. In the questionnaire, each of the eleven domains is specified through three items (except ‘emotions’, which is specified through four items) (see Appendix A). Consistent with previous questionnaires, the participants were asked to rate their level of agreement with each of the 34 items or statements on a seven-point scale (1 = strongly disagree; 7 = strongly agree). In addition to questions about the domains, sociodemographic and job information was collected: sex (male, female), age (in years), education (university degree yes/no), state, population of the community (number of inhabitants), profession (nurse, consultant etc.), extent of employment (full-time, part-time, voluntary), work experience (in years), proportion of content (CRs of PWD) related tasks within the last two years (in per cent), implementation of support services for CRs of PWD within the last two years (yes/no), importance of content (CRs of PWD) for the field of work and personal importance (each on a on a seven-point ratio scale (1 = no importance at all; 7 = very great importance). The questionnaire was subsequently pretested by ten CAs from different fields of work in the community and adjusted accordingly. After seven subsequent pilot tests, we did not obtain any new information and reached saturation. The CAs completed the questionnaire within 10–15 min on average. Final amendments were adopted in the questionnaire. The CIBQ was designed as an anonymous and voluntary online survey without any incentives for completion. For the data collection, the online survey application LimeSurvey (version 3.22.1 + 200129, LimeSurvey, Hamburg, Germany) was used [39].

### 2.3. Study Population and Data Collection

The study population consisted of actors and stakeholders from different communities in Germany. These actors were either already working with CRs and/or PWD or could potentially work with them. They were from different fields of work in the community, such as municipal administration, nursing, healthcare, voluntary work, consulting, church, sports, culture, education and housing. Only people of legal age (≥18 years) were authorised to participate in the survey.

Data were collected between October and December 2021. The CAs received an invitation and participation link via e-mail. In the case of non-response, the CAs received an e-mail reminder. A maximum of two reminder e-mails were sent at intervals of on average 10.5 working days. A total of 205 questionnaires were distributed to CAs who met the inclusion criteria. A total of 23 CAs declined to participate, and 182 CAs completed the questionnaire (88.78% response rate) and were included in our study.

Data were collected anonymously to reduce the potential of social desirability bias. The participating CAs were informed about the study and its conditions in writing before the survey started. Starting the online survey was only possible by accepting the data protection agreements. Withdrawal from the survey was possible at any time without giving a reason.

The dataset will be provided by the corresponding author on reasonable request.

### 2.4. Data Analysis

The data were analysed using the open-source software R (packages: sjPlot [40], MVN [41], psych [42], lavaan [43], semPlot [44]). We conducted all of the tests using 95% confidence with α = 0.05. If missing data was identified during the analysis, the whole case was left out of the analysis.

#### 2.4.1. Internal Consistency

The internal consistency of the eleven domains was assessed by using Cronbach’s alpha and the inter-item correlation. A Cronbach’s alpha between 0.70 and 0.95 is considered acceptable [45]. The value of the inter-item correlation should be ≥0.15–0.50 [46,47].

#### 2.4.2. Construct Validity

Before approving internal consistency and construct validity, the linearity and the multivariate normal distribution of the data were checked. These are preconditions to conduct a confirmatory factor analysis (CFA) [45]. The CFA was chosen for assessing the construct validity of the CIBQ because it is suitable for verifying the correct assignment of domains and items if these have already been assigned in advance on the basis of other questionnaires or the literature [45,48]. Therefore, each item should load on only one domain [48]. The CFA was performed with the lavaan package of R by using the maximum likelihood estimation (ML) or, more precisely, a robust version of the ML (MLR) to ensure that even if the data did not follow a normal distribution, they would be calculated and interpreted correctly [45,49]. If there were inappropriate items in the questionnaire, they could be removed to achieve better consistency and reduce the participants’ burden.

Next, inferential statistical tests were performed to assess the goodness-of-fit of structural equation models, such as the chi-square test [50]. Descriptive goodness-of-fit measures were reported because they allow for a gradual assessment of the deviation between the model and data [45]. The following guidelines for testing the model fit were considered:

- Chi-square to degrees of freedom ratio (CMIN/DF) < 2.00: The chi-square test statistic is generally regarded as being too stringent; therefore, the chi-square to degrees of freedom ratio was considered [51].
- Descriptive goodness-of-fit:
  - Standardised root mean square residual (SRMR) between ≤0.05 and ≤0.08: Absolute measure—average residual between standardised variables; is not based on the chi-square test [52,53].
  - Root mean square error of approximation (RMSEA) between ≤0.05 and ≤0.08: Parsimony index—measure of approximated fit [52,53].
  - Comparative fit index (CFI) ≥0.9: Incremental index—comparison of the examined model with the independence model [53,54].

Structural equation models can be plotted in the form of path diagrams [45]. To visualise the affiliation of the different items to the respective domains, a plot path diagram was created using the semPaths command in the lavaan package of R [42,43,44,55]. This function enables the graphical representation of the CFA.

## 3. Results

The characteristics of the study population are shown in Table 2.

The final sample comprised 182 CAs who participated in the online survey and completed the CIBQ. Of the CAs, 70.8% (n = 119) were female, and the mean age was 54.40 years (SD ± 11.09 years). The study participants had different levels of education: 15.8% (n = 26) had completed a training, 40.6% (n = 67) had a degree from a university of applied sciences, 32.7% (n = 54) had a university degree and 10.9% (n = 18) had a PhD. The CAs worked in various occupational fields. Nearly one-quarter (24.4%; n = 40) were active in counselling for senior citizens. The majority (82.3%; n = 139) worked full-time in the field of CRs and/or PWD. More than half (55.6%; n = 94) of the participants had been working with CRs and/or PWD for more than ten years, but less than 10% (6.1%; n = 10) were primarily concerned with this topic. On a scale from one (no importance at all) to seven (very great importance), the participants rated the importance of the topic of CRs of PWD as 4.22 (SD ± 1.90) for the respective occupational fields of the CAs and 4.83 (SD ± 1.77) for the CAs personally.

Most of the actors worked in Baden-Wuerttemberg (41.4%; n = 70), and CAs from seven of the sixteen states in Germany were represented. The represented municipal communities were small, middle or large cities according to the number of inhabitants. The majority (47.0%; n = 79) of CAs worked in medium-sized municipal communities (20,000 < 50,000 inhabitants). Only one participant (0.6%) described his community as having more than 500,000 inhabitants.

### 3.1. Internal Consistency

The Cronbach’s alpha and the inter-item correlations are shown in Table 3 and Table 4.

Cronbach’s alpha ranged from 0.56 to 0.89, and the inter-item correlation ranged from 0.08 to 0.88. Ten of the eleven domains showed internal consistency with a Cronbach’s alpha between 0.70 and 0.95 and an inter-item correlation of ≥0.15–0.50. Only one domain had a Cronbach’s alpha of 0.56 and an inter-item correlation of 0.08 (sociopolictical context). The underlying cause of dissatisfactory values was checked, and the responsible items D7b and D7c were excluded from further analysis. Accordingly, the CIBQ consisted of 32 items rather than 34, and the domain D7 sociopolitical context was reduced from three items to one item. As it is not possible to determine Cronbach’s alpha and the inter-item correlation within a domain containing only one item, the entire domain D7 sociopolitical context was also excluded from further analysis.

### 3.2. Construct Validity

**Figure 2:**
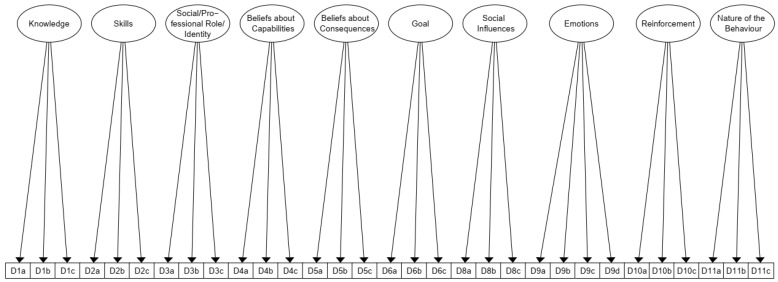
Path diagram of the CFA of the CIBQ. CFA = Confirmatory Factor Analysis; CIBQ = Community Implementation Behaviour. Circular nodes represent the latent variables (domains) whereas square nodes represent the manifest variables (items). The arrow represents a directed effect between a latent and a manifest variable.

The initial CFA with D7 showed the following model fit: CMIN/DF = 1.70; SRMR = 0.09; RMSEA = 0.07; CFI = 0.90. However, a SRMR of 0.09 is not satisfactory. A revised CFA without D7 showed the following model fit and indicated satisfactory construct validity: CMIN/DF = 1.63; SRMR = 0.05; RMSEA = 0.07; CFI = 0.92. Figure 2 shows the CFA, or more precisely, the affiliation of the items to the domains.

## 4. Discussion

In the investigated context, implementation depends not only on the behaviour and behaviour change in CAs but also on many other factors that facilitate or hinder the implementation of support services of CRs of PWD, such as participation of the target group, time, and money [15]. However, studies have shown that the implementation behaviour, or rather its change, is one essential way in which to optimise an implementation process that can solve many other problems or gaps in care [5].

The present study developed and tested the CIBQ for measuring the psychosocial domains of behaviour change among CAs for the implementation of support services for CRs of PWD in communities. The CIBQ is based on the original TDF and other research using the TDF. To the best of our knowledge, this is the first questionnaire to examine the necessary behaviour change among CAs to support CRs of PWD by considering the general gerontological and community contexts. Although there is a revised version of the TDF that comprises 14 domains [9], we decided to use the original version as a basis for our questionnaire [5] because studies have shown that the 12-domain version is more applicable for developing questionnaires [33]. Furthermore, the closely related DIBQ by Huijg et al. (2014) used this version [33]. While some frameworks and measurement tools for different areas of care already exist [32,34,35], the CIBQ can also be used for gerontological and community topics and to assess care gaps.

In contrast to most of the domains, the excluded domain sociopolitical context (D7) does not relate to the characteristics of the actors but to their environment [9]: (a) With the support of the federal government, the states and municipal communities, it is possible to implement support services for caring relatives of people with dementia in the community. (b) With the support of care insurance, it is possible to implement support services for caring relatives of people with dementia in the community. (c) With the given resources (e.g., staffing/funding), it is possible to implement support services for caring relatives of people with dementia in the community (see Appendix A). The items of D7 are very different and independent from each other and cover a large and diverse field using only three items. Furthermore, the participants have different relationships or connections to, for example, care insurance whereby volunteers may not be able to comment on care insurance at all, but carers would be familiar with this topic. This could be a possible explanation for the poor fit of D7, which led to the exclusion of this domain. The domain needs to be considerable adjusted if it is to be included.

The final questionnaire with ten domains and thirty-one items demonstrated good internal consistency and construct validity. In comparison to the validity and reliability of previous questionnaires, e.g., Taylor et al., 2013 (eleven domains; twenty-three items) [34] and Seward et al., 2017 (14 domains; 61 items) [35], also based on the TDF, the CIBQ showed similar values considering the CFA and the sufficient internal consistency (see Table 5). The chi-square test in both cases was significant (*p* value ≤0.05). Therefore, the chi-square to degrees of freedom ratio was also checked in the validation. The chi-square to degrees of freedom ratio of the CIBQ was better than those of Taylor et al. and Seward et al., and the CFI also had a better fit [34,35].

There are no official guidelines for supporting the CRs of PWD in municipal communities; however, such guidelines need to be implemented and realised, as is often the case in healthcare. Additionally, the implementation needs are much more individual than they are in health and thus cannot be generalised for the target group of CRs of PWD. The written context is interdisciplinary, similarly to the healthcare sector. However, in gerontology, there are many more actors and stakeholders who are in fields other than healthcare. This aspect makes it more difficult to specify a study population and the corresponding interventions or measures that need to be implemented. By analysing the relevant actors and enabling them to initiate necessary behavioural changes, the present study is a first step to optimise the implementation processes in this important and seminal field of caring for relatives of people with dementia. The CIBQ could be used by communities to determine their status quo and what they might work on in the future to optimise their implementation behaviours regarding caring relatives of people with dementia. For this purpose, however, the questionnaire should first be applied in science.

### Limitations

Although the results indicated internal consistency and construct validity of the CIBQ, there are some limitations that need to be taken into account. First, the recruitment of the study participants took place in the context of a cooperating project. Thus, the number of CAs was limited. A larger sample size would ensure more representative and confidential results. Future adaptations or further developments of the CIBQ should conduct a power calculation to ensure that the sample size is adequate to assess the number of variables, which would increase confidence in the results. Furthermore, the participants were not selected randomly but were identified through multipliers from the different communities. Thus, there is potential for selection bias, which needs to be considered for the content-related analysis of the CIBQ. Second, after testing for internal consistency, two items in one of the domains were excluded because of poor fitting. Therefore, one domain contains only one item. Less than three items per domain is not recommended [56]; hence, the entire domain D7 was excluded. In further research, the questions within D7 should be adapted so that this relevant domain can be included in the CIBQ.

## 5. Conclusions

To the best of our knowledge, the CIBQ is the first quantitative questionnaire that is applicable in the gerontological and municipal community context. The CIBQ serves as an appropriate way to understand the implementation behaviour of CAs and hence optimises the health and care of CRs of PWD, which might also positively affect the well-being of PWD. It is hoped that many researchers, practitioners, politicians, and CAs will use it. Initial results showed internal consistency and construct validity of the CIBQ for assessing factors and barriers related to the behavioural changes in CAs. Future research should apply the CIBQ to examine the barriers to behavioural change in CAs and use it to investigate additional community care gaps.

## Figures and Tables

**Table 1:** Definition of the domains and the adaptation to the gerontological context.

	Domain	Original Definition	Adaptation to the Gerontological Context
D1	Knowledge	An awareness of the existence of something.	The CAs have the knowledge about the situation and understand the relevance of CRs of PWD.
D2	Skills	An ability or proficiency acquired through practice.	The CAs have the skills and training to implement support services for CRs of PWD in the community.
D3	Social/Professional Role and Identity	A coherent set of behaviours and displayed personal qualities of an individual in a social or work setting.	The behavioural spectrum and the personal qualities of the CAs with regard to implementing support services for CRs of PWD in the community are part of the professional setting.
D4	Beliefs about Capabilities	Acceptance of the truth, reality or validity about an ability, talent, or faculty that a person can put to constructive use.	The CAs are confident in their abilities to implement support services for CRs of PWD in the community.
D5	Beliefs about Consequences	Acceptance of the truth, reality or validity about outcomes of a behaviour in a given situation.	The CAs understand the advantages and disadvantages of implementing support services for CRs of PWD in the community.
D6	Goals	Mental representations of outcomes or end states that an individual wants to achieve.	The CAs have goals that they would like to achieve regarding implementation of support services for CRs of PWD in the community.
D7 *	Sociopolitical Context	Any characteristics of the sociopolitical context that discourages or encourages the development of skills and abilities, independence, social competence, and adaptive behaviour.	The sociopolitical context has characteristics that motivate or discourage CAs to develop competences, skills, adaptive behaviours and independence regarding the implementation of support services for CRs of PWD in the community.
D8	Social Influences	Interpersonal processes that can cause individuals to change their thoughts, feelings, or behaviour.	Interpersonal processes that lead to a change in the CAs’ thoughts, feelings or actions regarding the implementation of support services for CRs of PWD in the community.
D9	Emotions	A complex positive/negative reaction pattern involving experiential, behavioural, and physiological elements by which the individual attempts to deal with a personally significant matter or event.	The CAs have positive emotions about implementing support services for CRs of PWD in the community.
D10	Reinforcement	Increasing the probability of a response by arranging a dependent relationship, or contingency, between the response and a given stimulus.	A given stimulus increases the probability of CAs implementing support services for CRs of PWD in the community.
D11	Nature of the Behaviour	The nature of the aggregate of all responses made by an individual in any situation.	The CAs have an original (intuitive, personal) way of behaving on which all actions and reactions in relation to CRs of PWD are based.

Domain definitions were based on definitions from Huijg et al. (2014) [33]. CA = Actor from the community; CR = Caring relative; PWD = Person with dementia. * D7 was excluded because of the poor fitting indicated by Cronbach’s alpha and the inter-item correlation.

**Table 2:** Characteristics of CAs.

Characteristics ^a^ (n = 182)	Total ^b^
Sex	
Female	70.8 (119)
Male	29.2 (49)
Age	54.40 ± 11.09
Education ^c^	
University of Applied Sciences Degree	40.6 (67)
University Degree	32.7 (54)
Training	15.8 (26)
PhD	10.9 (18)
Occupational field	
Consulting	24.4 (40)
Municipal administration-seniors’ work	17.1 (28)
Volunteering	14.0 (23)
Politics	10.4 (17)
Education	7.9 (13)
Medicine	6.7 (11)
Church	6.1 (10)
Nursing	4.9 (8)
Sports	4.3 (7)
Pharmacy	1.8 (3)
Culture	1.8 (3)
Living	0.6 (1)
Extent of employment in the field of CRs/PWD	
full-time	82.2 (139)
voluntary	17.8 (30)
part-time	0.0 (0)
Years of work in the field of CRs/PWD	
>10	55.6 (94)
>5–10	18.3 (31)
>2–5	16.6 (28)
0–2	9.5 (16)
Workload in the field of CRs/PWD	
<50%	93.9 (154)
>50%	6.1 (10)
Importance of CRs/PWD	
Personal (CAs)	4.83 ± 1.77
Occupational field	4.22 ± 1.90
State ^d^	
Baden-Wuerttemberg	41.4 (70)
Schleswig-Holstein	17.8 (30)
Hesse	17.2 (29)
North Rhine-Westphalia	6.5 (11)
Bavaria	5.9 (10)
Rhineland-Palatinate	5.9 (10)
Saxony-Anhalt	5.3 (9)
Number of inhabitants ^d^	
5.000 < 10.000	1.8 (3)
10.000 < 20.000	9.5 (16)
20.000 < 50.000	47.0 (79)
50.000 < 100.000	13.7 (23)
100.000–500.000	27.4 (46)
>500.000	0.6 (1)

^a^ Data presented as percentage (number) except for age and importance of CRs/PWD presented as mean ± standard deviation. ^b^ Data were missing for sex (n = 14); age (n = 11); education (n = 17); occupational field (n = 18); extent of employment in the field of CRs/PWD (n = 13); years of work in the field of CRs/PWD (n = 13); workload in the field of CRs/PWD (n = 18); importance of CRs/PWD occupational field (n = 15); importance of CRs/PWD personal (CAs) (n = 19); state (n = 13); number of inhabitants (n = 14). ^c^ The education corresponds to the German education system and has been arranged accordingly. ^d^ No CAs from: Berlin, Brandenburg, Bremen, Hamburg, Lower Saxony, Mecklenburg-Western Pomerania, Saarland, Saxony, Thuringia.

**Table 3:** Cronbach’s Alpha of each domain.

Domain	Number of Items	Cronbach’s Alpha
D1 Knowledge	3	0.74
D2 Skills	3	0.83
D3 Social/ Professional Role and Identity	3	0.88
D4 Beliefs about Capabilities	3	0.89
D5 Beliefs about Consequences	3	0.83
D6 Goals	3	0.89
D7 Sociopolitical Context *	3	0.56
D8 Social Influences	3	0.81
D9 Emotions	4	0.89
D10 Reinforcement	3	0.84
D11 Nature of Behaviour	3	0.89

* D7 needs to be excluded for further analysis because of unfitting values in Cronbach’s Alpha and Inter-Item Correlation.

**Table 4:** Inter-Item Correlation.

Item	Inter-Item Correlation
D1 Knowledge	
D1a & D1b	0.48
D1a & D1c	0.38
D1b & D1c	0.60
D2 Skills	
D2a & D2b	0.57
D2a & D2c	0.53
D2b & D2c	0.74
D3 Social/ Professional Role and Identity	
D3a & D3b	0.75
D3a & D3c	0.71
D3b & D3c	0.65
D4 Beliefs about Capabilities	
D4a & D4b	0.88
D4a & D4c	0.63
D4b & D4c	0.68
D5 Beliefs about Consequences	
D5a & D5b	0.68
D5a & D5c	0.56
D5b & D5c	0.65
D6 Goals	
D6a & D6b	0.66
D6a & D6c	0.73
D6b & D6c	0.77
D7 Socio-Political Context *	
D7a & D7b	0.73
D7a & D7c	0.08
D7b & D7c	0.08
D8 Social Influences	
D8a & D8b	0.52
D8a & D8c	0.59
D8b & D8c	0.66
D9 Emotions	
D9a & D9b	0.75
D9a & D9c	0.60
D9a & D9d	0.58
D9b & D9c	0.68
D9b & D9d	0.60
D9c & D9d	0.77
D10 Reinforcement	
D10a & D10b	0.56
D10a & D10c	0.57
D10b & D10c	0.76
D11 Nature of Behaviour	
D11a & D11b	0.73
D11a & D11c	0.72
D11b & D11c	0.77

* D7 needs to be excluded for further analysis because of poor fitting indicated by Cronbach’s alpha and inter-item correlation.

**Table 5:** Comparison of construct validity and internal consistency of different questionnaires.

	CIBQ *	Taylor et al., 2013 [34]	Seward et al., 2017 [35]
CFA			
CMIN/DF	1.63	1.98	2.5
SRMR	0.05	-	0.07
RMSEA	0.07	0.06	0.07
CFI	0.92	-	0.78
Internal Consistency			
Cronbach’s Alpha	0.74–0.89	-	0.61–0.90
Inter-Item correlation	0.38–0.88	0.21–0.64	-

* Including ten out of initially eleven domains. CIBQ = Community Implementation Behaviour; CFA = Confirmatory Factor Analysis; CMIN/DF = Chi-square to degrees of freedom ratio; SRMR = Standardised root mean square residual; RMSEA = Root mean square error of approximation; CFI = Comparative fit index.

## Data Availability

The datasets used and/or analysed during the current study are available from the corresponding author on reasonable request.

## Appendix A

**Table A1:** Community Implementation Behaviour Questionnaire ^a^.

	Domain	Items
D1	Knowledge	a. I know why it is important to implement support services for CRs ^b^ of PWD ^c^ in the community. b. I know the goals for the implementation of support services for CRs of PWD in the community. c. I know what is expected of me in the implementation of support services for CRs of PWD in the community.
D2	Skills	a. I have been trained (e.g. in the context of training/ further training/ induction etc.) on the implementation of support services for CRs of PWD in the community. b. I have the skills or the necessary knowledge to implement support services CRs of PWD in the community. c. I am experienced in implementing support services for CRs of PWD in the community.
D3	Social/Professional Role and Identity	a. The implementation of support services for CRs of PWD in the community is part of my job description and my area of responsibility. b. I see it as part of my remit to implement support services for CRs of PWD in the community. c. My role in the implementation of support services for CRs of PWD in the community is clearly defined for me.
D4	Beliefs about Capabilities	a. I am convinced that I can implement support services for CRs of PWD in the community. b. I am convinced that I can implement support services for CRs of PWD in the community, even if obstacles arise. c. I am convinced that I can implement support services for CRs of PWD in the community, even if CRs of PWD are not motivated.
D5	Beliefs about Consequences	a. If I implement support services for CRs of PWD in the community, the cooperation in my work environment is strengthened. b. The implementation of support services for CRs of PWD in the community is a satisfying task for me. c. When I implement support services for CRs of PWD in the community, it helps CRs of PWD to be better cared for.
D6	Goals	a. Within the scope of my field of activity, one of my goals is the implementation of support services for CRs of PWD in the community. b. I set myself realistic short-term goals regarding the implementation of support services for CRs of PWD in the community. c. I set myself realistic long-term goals regarding the implementation of support services for CRs of PWD in the community.
D7 ^b^	Sociopolitical Context	a. With the support of the federal government, the states and municipal communities it is possible to implement support services for CRs of PWD in the community. b. With the support of the care insurance it is possible to implement support services for CRs of PWD in the community. c. With the (given) resources (e.g. staffing/funding) it is possible to implement support services for CRs of PWD in the community.
D8	Social Influences	a. Most of the people I matter about are in favour of me implementing support services for CRs of PWD in the community. b. People from my work environment (also) implement support services for CRs of PWD in the community. c. People from my work environment are helpful in implementing support services for CRs of PWD in the community.
D9	Emotions	a. When I implement support services for CRs of PWD in the community, I am optimistic. b. When I implement support services for CRs of PWD in the community, I feel comfortable. c. When I implement support services for CRs of PWD in the community, I am not insecure. d. When I implement support services for CRs of PWD in the community, I am not frustrated.
D10	Reinforcement	a. When I implement support services for CRs of PWD in the community, it is valued by carers of people with dementia. b. When I implement support services for CRs of PWD in the community, I receive recognition from my work environment. c. When I implement support services for CRs of PWD in the community, I receive recognition from my private environment.
D11	Nature of the Behaviour	a. The implementation of support services for CRs of PWD in the community is something that comes naturally to me (personally). b. The implementation of support services for CRs of PWD in the community is something that I pursue on my own initiative (also independent of my job description/area of responsibility). c. The implementation of support services for CRs of PWD in the community is important for me.

Domain definitions were based on definitions from Huijg et al. [33]. ^a^ The questionnaire was developed in German. For the purpose of this article, all items were translated into English. ^b^ CRs = Caring relatives. ^c^ PWD = People with dementia.
